# Psychometric validation of the visual function questionnaire-25 in patients with diabetic macular edema

**DOI:** 10.1186/1477-7525-11-10

**Published:** 2013-01-24

**Authors:** Andrew J Lloyd, Jane Loftus, Michelle Turner, Ginny Lai, Andreas Pleil

**Affiliations:** 1 Oxford Outcomes, an ICON plc Company, Seacourt Tower, West Way, Oxford, OX2 0JJ, UK; 2 Pfizer Ltd, Walton Oaks, Dorking Road, Tadworth, Surrey, KT20 7NS, UK; 3 ICON PLC, Embarcadero Centre, San Francisco, CA, USA; 4 Pfizer Inc, La Jolla, CA, USA

**Keywords:** Diabetic macular edema, Visual functioning questionnaire, Psychometric analyses

## Abstract

**Background:**

Diabetic Macular Edema (DME) is a common cause of impaired vision and blindness amongst diabetics. If not detected and treated early, the resulting vision loss can lead to considerable health costs and decreased health-related quality of life (HRQoL). The aim of this study was to provide evidence of the psychometric properties of the National Eye Institute - Visual Functioning Questionnaire (VFQ-25) for use in a cohort of DME patients who participated in a clinical efficacy and safety trial of pegaptinib sodium (Macugen).

**Methods:**

A phase 2/3 randomised, double masked trial evaluated pegaptanib injection versus sham injection in patients with DME. The analysis was conducted using baseline HRQoL data of the VFQ-25 and the EQ-5D, on a modified intent-to-treat sample of 235 patients. These measures were administered by a trained interviewer by telephone in all but one of the study countries, where face-to-face interviews were conducted in the clinic. The measures were completed in the week prior to baseline, and after 54 weeks of treatment. Distance visual acuity, measured according to the Early Treatment Diabetic Retinopathy Study (ETDRS), was assessed at all time points. Psychometric properties of the VFQ-25 assessed included domain structure, reliability, concurrent and construct validity, responsiveness.

**Results:**

The VFQ-25 was found to consist of 11 domains slightly different than those proposed. Nevertheless, none of the eight established multi-item scales met the criterion for further splitting and the VFQ-25 was scored as in the developers’ instructions. Internal consistency reliability was demonstrated for six out of the eight original multi-item scales, with Cronbach's alpha ranging from 0.58 (Distance Activities) to 0.85 (Vision Specific: Dependency). The VFQ-25 domains generally showed a low to moderate correlation with EQ-5D visual analogue scale (range 0.16-0.43) and with the visual acuity score (range 0.10-0.41). Construct validity was upheld with higher VFQ-25 scores for patients who saw more letters according to the ETDRS. Almost all scales were shown to be responsive with Guyatt's statistic ranging from 0.10 to 0.56 at 54 weeks.

**Conclusions:**

The VFQ-25 has evidence to support its validity and reliability for measuring HRQoL in DME. However, some operating characteristics of the instrument need further consideration and discussion in the case of DME patients. Further research is therefore warranted in this indication.

## Background

Diabetic macular edema (DME) is one of the most common causes of impaired vision and blindness amongst diabetic patients [1]. DME is the result of long-term excess blood-glucose on the microvascular system of the eye. Over time, this leads to diabetic retinopathy, and in some patients results in edema of the macula (the cone-rich centre of the retina responsible for day-time colour vision). The Early Treatment Diabetic Retinopathy Study (ETDRS), a pivotal study in DME, defined macular edema as thickening of the retina and/or hard exudates within one disc diameter of the centre of the macula [2]. The standard of care to treat DME is focal/grid laser photocoagulation; however, earlier in 2011 intravitral injection of an anti-vascular endothelial growth factor, ranibizumab, was approved for the treatment of DME, and further treatments are in development.

In addition to visual improvement, it is important to measure efficacy of DME treatment which is evaluated in terms of the impact on patients’ ability to function and complete their usual activities. Measures of vision-related or health-related quality of life (HRQoL) have been developed to assess the broader impact of poor visual functioning [3,4]. Generic measures such as the EQ-5D also exist which are designed to be valid assessments of HRQoL in any disease area [5].

Decreasing vision has a complex relationship with HRQoL; in addition to the impact on people’s ability to complete their usual daily activities, the loss of visual function combined with the threat of further declines in visual function may affect psychological state or lead to social isolation [6]. There are several condition-specific measures or instruments which are designed to capture the specific impact of vision loss on HRQoL. The National Eye Institute in collaboration with RAND developed the Visual Function Questionnaire (VFQ-25), which is probably the most widely used and researched HRQoL measure in this field. The VFQ-25 is a vision-specific measure of HRQoL composed of eight multi-item scales, four single-item scales, and one composite score ranging in value from 0 (poor) to 100 (high HRQoL). The following dimensions of vision-targeted function and HRQoL comprise the VFQ-25: overall vision, difficulty with near vision activities, difficulty with distance vision activities, limitations in social functioning due to vision, role limitations due to vision, dependency on others due to vision, mental health symptoms due to vision, driving difficulties, limitations with peripheral and colour vision, and ocular pain. The measure provides an algorithm to calculate a composite score based on the subscales, and additionally contains one item for subject rating of general health status.

The development and psychometric characteristics of the VFQ-25 have been documented [7,8]. The VFQ-25 has been developed to be appropriate to use across a range of visual disorders and to measure their effects. This is an advantage over other measures which are more specific such as the VF-14 [4] which was developed to assess outcomes associated with cataracts and associated treatments. The VFQ-25 has been used in a wide range of different ophthalmology indications [9]. The Los Angeles Latino Eye Study (LALES) is one prominent example where the impact of vision loss on HRQoL was assessed over four years in a population cohort. The study assessed different conditions including glaucoma, retinopathy and age-related macular degeneration. This work revealed the domains of the VFQ-25, such as vision-related mental health, which were most sensitive to loss of vision. Mazhar et al. [10] present more LALES data which focussed on the changes in HRQoL experienced by people with diabetic retinopathy.

Existing evidence regarding the VFQ-25 in other indications suggests that it possesses good internal consistency reliability, and correlates well overall with visual acuity (measured in terms of ETDRS) [7,8]. Despite the widespread use of the VFQ-25, the evidence of validity of the VFQ-25 in people with DME is sparse and needs further elucidation. Psychometric validation of instruments should always be considered as an on-going process [11], and the validity of an instrument can never be assumed just because it has been demonstrated in a related area. Therefore it is very important to measure and establish the psychometric properties of an instrument when used in a new indication. This is standard practice in outcomes research and is the expectation of regulatory bodies and decision makers [11]. Vision disorders in diabetes are known to affect HRQoL [12]. Lloyd et al., 2008 [13], and Cusick et al. (2005) [6] show evidence regarding how edema exerts an additional burden on HRQoL. As the psychometric properties of the VFQ-25 have not been documented in this patient group, the present study is designed to test the psychometric properties of the VFQ-25 in an adult DME population using data collected during a clinical trial of the treatment of pegaptanib for DME.

## Methods

### Data & participants

The data from this trial included patients who were randomised to either intravitreous (IVT) pegaptanib or sham treatment [14,15]. In brief, the pivotal study was a multicenter, randomized, sham-controlled, double-masked, parallel-group, comparative trial to confirm the safety and efficacy of pegaptanib sodium 0.3 mg, when given as IVT injections versus sham injections. Only one eye per patient was treated and this could be either the worse or better eye, depending on the decision of the treating physician. At Week 18, both arms of the trial could receive focal/grid laser photocoagulation at the discretion of the investigator according to ETDRS criteria. Subsequently patients could receive focal/grid laser photocoagulation, provided that a minimum of 17 weeks had elapsed between treatments. Patients could not receive more than three focal/grid photocoagulations per year. The primary efficacy endpoint in the trial was the proportion of patients who experienced a greater than or equal to ten letter (or 2 line) improvement in vision (ETDRS) from baseline at 1 year.

Patients were enrolled at sites around the world including Australia, Canada, Europe, India, South Africa and South America. The sample used for the psychometric analyses included the modified intention to treat Year 1 (MITT1) population which included all randomized subjects who received at least one dose of study medication, who completed the baseline visual acuity assessment, and who had at least one post-baseline visual acuity assessment by Week 54. In addition to the VFQ-25, the EQ-5D questionnaire was administered to patients at Baseline, Week 18, Year 1, Year 2, and also to any early withdrawals. The study results from the HRQoL assessments have been reported elsewhere [15].

### Measures

The validated and translated interview versions of the VFQ-25 were administered to patients in this study. Trained, certified call centre operatives telephoned all patients in their homes and administered the questionnaires in the patient’s native language. The exception to this was the centres in India, whereby the questionnaires were completed face-to-face in an interview setting at the clinic. This was necessary because there was a lack of available speakers of the required five Indian languages at the call centre, and there was difficulty in ensuring all patients had telephone access. Prior to randomization, all patients were supplied with the patient brochures and interview guides to assist them with completing the study questionnaires. This included the instructions and the response categories as a reference to use during the interview if they preferred. Standard scoring procedures were used [8].

The EQ-5D is a generic preference weighted HRQoL measure often used to support the estimation of quality-adjusted life years for use in Health Technology Assessments. It assesses health status in five domains (mobility, self-care, usual activities, pain/discomfort and anxiety/depression) that are transformed to a single index score, and also includes a visual analogue scale (VAS) for self-rated health status [5,16]. Only the EQ-5D VAS was used for analyses in this study. Scores for the VAS range from 0–100 where 100 represents the best imaginable health, and 0 represents worst imaginable health.

### Analyses

Analyses were conducted on the MITT1 sample, using both baseline and 54-week data. All analyses were conducted irrespective of the assignment to treatment arm (i.e. on masked data). All analyses were described in a statistical analysis plan (SAP) which was finalised prior to initiating any analysis. The psychometric properties of the VFQ-25 were evaluated using standard tests and criteria [11,17,18]. These included an examination of item and scale variability, domain structure, internal consistency reliability, item-total correlation, concurrent validity, construct validity, responsiveness, and an estimate of MID. The level of missing data and the frequency of floor effects (number of respondents with the lowest possible score) and ceiling effects (number of respondents with the highest possible score) were also reviewed for items and scales. All analyses were conducted in SAS Version 9.1.3 for Windows and the SAS code was independently reviewed.

### Conceptual framework

The relationship between items and domains in the questionnaire, referred to as the conceptual framework, was explored using principal components analysis. Principal components analysis attempts to reduce the items (questions) into a number of smaller components and was undertaken here to determine whether the items loaded into the pattern that was hypothesised by the instrument developers. Models were attempted which used the eigenvalue >1 as one selection criteria. In addition alternative models were also developed which forced certain factor solutions. Orthogonal (varimax) and oblique (promax) rotations were applied in an attempt to provide a model that best described the data, and variable (item) clustering methods were used to confirm the scale structure and test the robustness of the resulting multi-item scales [19]. Variable cluster analysis is a form of factor analysis that offers interpretative advantages by identifying groups of variables (items) that are as correlated to each other among themselves and uncorrelated to other groups as possible [20]; it was undertaken by inputting a polychoric correlation matrix into SAS PROC VARCLUS, with each item group (cluster) being split into two dimensions until each remaining cluster had a second eigenvalue of less than one [19].

### Reliability

Reliability is the extent to which a scale score is associated with random error. It is evaluated to ensure that the measure produces stable and internally consistent scores. Internal consistency and reliability was measured by Cronbach’s α [21], which identifies the degree to which items within a scale correlate with each other to constitute a multi-item scale. An α coefficient ≥0.70 is considered acceptable [22]. Based on the available trial data there was no opportunity to estimate test-retest reliability.

Item - total (score) correlations indicate the degree of homogeneity within a scale. Items are expected to correlate more highly with their domain score than with any other domain score [17,23]. Item-total correlation was evaluated using the Pearson correlation coefficient. It was not possible to estimate this metric for subscales on the VFQ-25 with only one item (e.g. colour vision). Despite this, the SAP stated that items should correlate more highly with the domain score than other domains or sub-scale scores [23].

### Validity

Concurrent validity was assessed by comparing subscale scores of the VFQ-25 with the EQ-5D VAS score (the only other self-assessed rating of HRQoL in the study). The other single index score from the EQ-5D, which is based on five dimensions of health, was not included in the concurrent validity partly because the scores are preference weighted, and because of evidence that the EQ-5D single index score has been shown to be insensitive to visual impairment [24].

It is difficult to make firm predictions regarding the nature of the relationship between VFQ-25 and EQ-5D VAS because they are not conceptually measuring the same thing. The VFQ-25 is a disease specific measure of HRQoL which focuses heavily on the impact of visual impairment. The EQ-5D VAS is a measure of self-rated health which reflects a totally subjective assessment of the quality of their current health state and is subject to many influences beyond vision. Therefore, there are *a priori* reasons to believe that the degree of association between these measures will be moderate at best. It was hypothesised therefore in the SAP that correlation coefficients between EQ-5D VAS and VFQ-25 domain scores may not be much greater than 0.30. Total number of letters (on ETDRS) of the patient’s visual acuity at 4 m and 1 m were also included as quasi-continuous variables in the correlation analyses of concurrent validity. Correlations with both the study eye and the fellow eye were estimated because patients’ self-report of their HRQoL and how their visual function affects them will be affected by both eyes. Previous research has shown that HRQoL can improve, regardless of whether the treated eye is better or worse than the seeing eye [25,26].

Construct validity, whether the items/domains conform to what is predicted by the conceptual framework, was measured using both discriminant (known groups) and convergent (strength of association) approaches. Known groups validity was assessed by comparing scores of groups of patients defined in terms of ETDRS visual acuity with analysis of variance and additional post hoc tests (Tukey’s test). Convergent validity was assessed using correlation and multiple regression analyses to explore the relationship between VFQ-25 and other disease-related variables. Data were analysed by calculating correlation coefficients between VFQ-25 domain scores and ETDRS scores and by fitting step-wise multiple linear regression models to determine the predictors of VFQ-25 domain scores. The models included vision-related factors: ETDRS visual acuity and duration of vision problems, diabetes-related variables (including measurement of glycated haemoglobin (HbA1c) and presence of other diabetic complications), and background characteristics such as age and sex, Eastern Cooperative Oncology Group (ECOG) status, and smoking status. It was predicted that the VFQ-25 will be statistically associated with both vision specific-variables and diabetes-related variables.

### Sensitivity

Sensitivity to change, or responsiveness, is the ability of an instrument to reflect objective changes in a population. Sensitivity was assessed in terms of changes reported by those patients with at least a five letter improvement in visual acuity. In many studies, a clinically relevant improvement is defined as ten letters at one year. The use of five letters in the present analysis was used as a stricter test and has been used as a benchmark in other recent research [27]. Additionally, the use of five letters as the criterion meant that data were available from more patients to test sensitivity. Among patients with a ≥5 letter improvement in the study eye between baseline and week 54, sensitivity of the VFQ-25 was evaluated in terms of effect size [28], standardized response mean [29], Guyatt’s responsiveness statistic [30], and the significance of the mean change from baseline to week 54 assessed by the one-sample *t*-test. The effect size is the mean change score divided by the SD of the baseline score. An interpretation of effect size in the context of analysis of variance has been provided in terms of benchmarks where 0.1 is a small effect, 0.25 is a medium effect and 0.4 is a large effect [31]. The standardized response mean is the mean change score divided by the SD of the change score. Guyatt’s responsiveness statistic is the mean change score divided by the SD of the change in subjects who remained stable as defined by an increase or decrease of only up to 1 letter. A Guyatt’s statistic of > .20 is considered acceptable. Change over time was compared for those who showed a response to treatment.

### Minimal important difference

MID is used to interpret whether the observed change is important from a patient’s perspective. The MID of the VFQ-25 was assessed using two distribution based approaches [30,32]. The first is a one standard error of measurement (SEM) to reflect a MID in individual patient scores. The SEM reflects the SD of the change from baseline to week 54 and the test psychometric reliability (estimated as a SD x square root of (1- reliability score)). A confidence interval (CI) can be calculated around a test score using the SEM to indicate the smallest amount of change that would be expected to occur in the individual with the appropriate degree of confidence/probability. This interval is often termed the minimal detectable change (MDC), which for a 95% CI would be an MDC_95_. Pickard et al. has estimated the MID on the EQ-5D VAS to be 7 [33]. The degree of change between baseline and year 1 on all subscales of the VFQ-25 was estimated for patients who report a change of 7 on EQ-5D VAS. No formal assessments of self-reported change or ‘anchor questions’ specific to the domains of the VFQ-25 were available for assessing MID.

## Results

**Figure 1:**
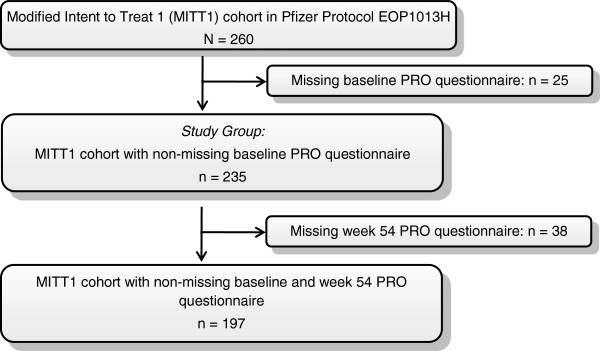
CONSORT diagram.

From this analysis cohort of 260 patients, 235 had non-missing VFQ-25 baseline data and were included in the psychometric validation (Figure 1). Table 1 shows the background characteristics of the study group. The mean ± SD age of the analysis cohort was 62 ± 10 years, 43% were female, and 82% were Caucasian. At baseline the majority of the patients (68%) had visual acuity in the study eye of 69 to 55 ETDRS letters (20/50 to 20/80). Data are also presented for patients in terms of better eye and worse eye visual acuity.

**Table 1:** Background characteristics of study group

**Characteristic**	**Baseline (week 0) N = 235**
Age (years)	
Mean ± SD	62 ± 10
Median (IQR)	62 (56 – 69)
Range	20 – 83
Female, n (%)	102 (43.4)
Ethnicity, n (%)	
Caucasian/White	192 (81.7)
Asian	25 (10.6)
Other (Black, Hispanic/Latino, unspecified)	18 (7.6)
ECOG Performance Status	
Mean ± SD	0.5 ± 0.5
Median (IQR)	0 (0 – 1)
Range	0 – 2
Type II diabetes, n (%)	218 (92.8)
Duration of vision problems, n (%)	
None	1 (0.4)
<1 year	93 (39.6)
1-5 years	122 (51.9)
6-10 years	15 (6.4)
>11 years	4 (1.7)
ETDRS visual acuity scale: study eye	
85 letters or more (20/16 or more)	0 (0.0)
85-75 letters (20/20 - 20/32)	0 (0.0)
74-70 letters (20/40)	3 (1.3)
69-55 letters (20/50 - 20/80)	160 (68.1)
54-40 letters (20/100 - 20/160)	61 (26.0)
39-35 letters (20/200)	11 (4.7)
34 letters or less (20/250 or less)	0 (0.0)
ETDRS visual acuity scale: fellow eye	
85 letters or more (20/16 or more)	11 (4.7)
85-75 letters (20/20 - 20/32)	75 (32.2)
74-70 letters (20/40)	30 (12.9)
69-55 letters (20/50 - 20/80)	60 (25.8)
54-40 letters (20/100 - 20/160)	31 (13.3)
39-35 letters (20/200)	9 (3.9)
34 letters or less (20/250 or less)	14 (6.0)
Counting fingers-hand motion	3 (1.3)

* ETDRS = Early Treatment Diabetic Retinopathy Study.

Note: Percentages are based on non-missing categories.

With regard to item and scale variability, patients used the entire range of responses for all of the VFQ-25 items. In addition, the completion rate for all items on the VFQ-25 was 96% [26,34]. The distribution of the VFQ-25 data by scale at baseline and at week 54 is presented in Table 2. The VFQ-25 composite score at baseline was 66.8 ± 18.6. The smallest and largest means were found for General Health (40.2 ± 22.2) and Colour Vision (87.1 ± 21.2). Scores were slightly higher at week 54 on the majority of domains, although direct comparisons cannot be made between baseline and week 54 scores because the baseline cohort was not restricted to those with follow-up data.

**Table 2:** Distribution of scale scores

**Questionnaire and Domain**	**Question number(s)**	**Baseline (week 0) N = 235**		**Follow-up (week 54) N = 197**	
		**Mean ± SD**	**Median (IQR)**	**Mean ± SD**	**Median (IQR)**
VFQ-25: *Empirical range is 0 to 100 where a higher score indicates better HRQoL*					
1. General Health	1	40.2 ± 22.2	50.0 (25.0-50.0)	40.9 ± 21.0	50.0 (25.0-50.0)
2. General Vision	2	54.6 ± 17.8	60.0 (40.0-60.0)	62.2 ± 15.4	60.0 (60.0-80.0)
3. Ocular Pain	4, 19	78.8 ± 22.3	87.5 (62.5-100.0)	82.2 ± 19.7	87.5 (62.5-100.0)
4. Near Activities	5, 6, 7	58.6 ± 23.8	58.3 (33.3-75.0)	61.0 ± 23.9	58.3 (41.7-83.3)
5. Distance Activities	8, 9, 14	64.2 ± 24.4	66.7 (41.7-83.3)	66.1 ± 25.6	66.7 (50.0-91.7)
6. Social Functioning	11, 13	80.1 ± 23.1	87.5 (62.5-100.0)	78.6 ± 23.2	87.5 (62.5-100.0)
7. Mental Health	3, 21, 22, 25	58.2 ± 27.7	62.5 (37.5-81.3)	64.2 ± 25.2	68.8 (50.0-87.5)
8. Role Difficulties	17, 18	54.8 ± 28.0	50.0 (37.5-75.0)	61.7 ± 27.8	62.5 (37.5-87.5)
9. Dependence	20, 23, 24	70.1 ± 31.2	83.3 (41.7-100.0)	73.7 ± 28.2	83.3 (58.3-100.0)
10. Driving*	15c, 16, 16a	51.8 ± 34.9	58.3 (16.7-83.3)	53.9 ± 35.6	66.7 (16.7-83.3)
11. Colour Vision	12	87.1 ± 21.2	100.0 (75.0-100.0)	87.2 ± 21.5	100.0 (75.0-100.0)
12. Peripheral Vision	10	71.3 ± 25.8	75.0 (50.0-100.0)	74.6 ± 25.4	75.0 (50.0-100.0)
VFQ-25 Composite	1-14, 15c, 16, 16a, 17-25	66.8 ± 18.6	68.8 (53.5-82.8)	70.1 ± 18.7	71.5 (57.2-86.6)
EQ-5D VAS: *Empirical range is 0 to 100 where a higher score indicates better health*		65.7 ± 16.8	70 (50-80)	67.3 ± 17.0	70 (60-80)
EQ-5D single index		0.748 ± 0.203	0.779 (0.689-0.848)	0.727 ± 0.238	0.727 (0.689-0.850)

Note: Direct comparison between baseline and follow-up data should not be made because the baseline cohort was not restricted to those who also have follow-up data.

* Some patients are no longer currently driving, leading to higher amount of missingness in this subscale: N = 151 at baseline and N = 122 at week 54.

### Domain structure

Initial principal components analysis with varimax rotation identified a simple five factor solution (data not shown). The item loadings on the newly defined factors from the baseline dataset showed a reasonable consistency when one looked at the vision functioning and psychosocial components of the instrument, but less consistency when one looked at specific factors. In a forced 11 factor model (excluding General Health) using a varimax rotation, there was no significant item loading on the 11th factor. Of the remaining ten factors, three contained only one item and these showed a tendency to be part of other factors. Of the seven primary factors, one factor included seven of the 11 items from the psychosocial component of the original scales (i.e. mental health, social and role functioning, and dependency); the role difficulty scale (items 17 and 18) created its own factor in the same way as the original factor structure. The items in the vision functioning domains did not load identically. Of the 11 items in the original model, all but the colour vision item appeared to be represented, though not in the exact six domains. The findings for the analyses based on non-orthogonal rotations produced very similar patterns and so are not reported in detail here. Variable/item clustering methodology showed that none of the eight established multi-item scales met the criterion for further splitting.

### Reliability

Internal consistency reliability was demonstrated for six out of the eight VFQ-25 multi-item scales, with Cronbach's alpha ranging from 0.58 (Distance Activities) to 0.85 (Vision Specific: Dependency) (Table 3). The VFQ-25 composite score showed a Cronbach’s alpha of 0.92 at baseline. A Cronbach’s alpha of 0.70 or greater was achieved for each multi-item scale with the exception of Distance Activities and Social Functioning (0.64). It should be noted though that these two domains are based on just two items and so this will naturally limit any estimates of Cronbach’s alpha.

**Table 3:** Internal consistency reliability, concurrent validity, and construct validity at baseline

	**Internal-consistency reliability**	**Concurrent validity**			**Construct validity**		
		**EQ-5D VAS**	**Study eye: ETDRS total letters**	**Fellow eye: ETDRS Total letters**	**Study eye: ETDRS 73–64 letters**	**Study eye: ETDRS 52–35 letters**	
**VFQ-25 domain**	Cronbach’s alpha*	**Pearson correlation**			**Mean ± SD**		***P *value from student’s *t*-test**
1. General Health	N/A	0.43	0.19	0.10	41.0 ± 24.17	33.5 ± 22.07	.078
2. General Vision	N/A	0.30	0.31	0.29	60.3 ± 15.27	46.4 ± 15.95	<.001
3. Ocular Pain	0.70	0.20	0.10	0.01	78.9 ± 23.67	76.1 ± 24.49	.52
4. Near Activities	0.73	0.36	0.35	0.36	65.8 ± 21.93	45.1 ± 21.55	<.001
5. Distance Activities	0.58	0.33	0.34	0.36	70.9 ± 23.44	51.2 ± 23.99	<.001
6. Social Functioning	0.64	0.33	0.31	0.34	84.8 ± 19.51	69.1 ± 27.40	<.001
7. Mental Health	0.78	0.35	0.33	0.30	65.3 ± 24.96	44.8 ± 27.62	<.001
8. Role Difficulties	0.77	0.16	0.27	0.23	59.4 ± 30.67	44.1 ± 26.40	.004
9. Dependency	0.85	0.37	0.34	0.34	78.0 ± 26.40	55.6 ± 33.39	<.001
10. Driving	0.75	0.34	0.41	0.51	65.5 ± 32.09	31.8 ± 33.52	<.001
11. Colour Vision	N/A	0.19	0.18	0.14	87.7 ± 19.16	81.8 ± 24.49	.14
12. Peripheral Vision	N/A	0.19	0.26	0.36	75.4 ± 25.83	61.4 ± 25.14	.003
VFQ-25 Composite	0.92	0.38	0.39	0.40	72.1 ± 17.91	56.1 ± 18.00	<.001

* Cronbach’s alpha is only applicable for multi-item scales.

### Validity

Concurrent validity findings were mixed (Table 3). The VFQ-25 domains generally showed a low-to-moderate correlation with the EQ-5D VAS (range 0.16 – 0.43). There were particularly low correlations between EQ-5D VAS and Role Difficulties, Colour Vision, Peripheral Vision, and Ocular Pain. The slightly higher correlations (compared with the correlations with visual acuity) suggest that the VFQ-25 may be measuring HRQoL in a quite generic way.

Known groups validity was upheld with higher VFQ-25 scores for patients who saw more ETDRS letters. The mean VFQ-25 composite score was significantly higher when comparing the quartile of patients with the best vision to the quartile of patients with the worst vision (based on ETDRS visual acuity score) (72.1 ± 17.9 and 56.1 ± 18.0, respectively, P value < .001). With the exception of Ocular Pain and Colour Vision (P value = 0.52 and 0.14, respectively), all other results were statistically significant (Table 3).

Convergent validity findings were mixed. The domains overall showed low to moderate correlations with ETDRS visual acuity score for the study eye (range 0.10 – 0.41), and for the fellow eye (range 0.01 – 0.51). The domains of Role Difficulties, Colour Vision, Peripheral Vision, and Ocular Pain correlated poorly (<0.3) with the ETDRS visual acuity score. Convergent validity was further explored through multiple regression analyses (data not shown). Using step-wise selection, it was found that the ETDRS visual acuity, EQ-5D VAS, HbA1c, age, gender, ECOG and duration of vision problems were all predictors of different VFQ-25 domain scores (Table 4). EQ-5D VAS and ETDRS were the most consistent predictors of VFQ scores. These results also suggested that the VFQ-25 may be measuring HRQoL in a quite generic way, capturing many aspects of general health status rather than purely vision specific information.

**Table 4:** Results from stepwise multiple regression analysis of predictors of VFQ-25 domain scores

	**EQ-5D VAS**	**HbA1C <7.6%**	**ETDRS**	**Duration of vision problems**	**Age**	**Gender**	**ECOG**
General health	**	**	*				
General vision	**		**				
Ocular pain	**						
Near activities	**		**				
Distance activities	**		**				
Social functioning	**		**				
Mental health	**	*	**	*			
Role difficulties	*	**	*	*	*		
Dependency	**	*	**	*			
Driving	**	*	*			*	
Color vision	**						
Peripheral vision	**		**				*

* = *P* < 0.05, ** = *P* < 0.01

### Sensitivity

The data in Table 5 show the sensitivity to change of the instrument in patients who reported a ≥5 gain from baseline to Week 54. The mean change varied from 0.54 (Social Functioning) to 11.29 (Role Difficulties), with an overall change of 4.90 for the VFQ-25 composite score (P value < .001). However, only General Vision, Near Activities, Mental Health, Role Difficulties, and Driving showed statistically significant differences assessed by the one-sample *t*-test.

**Table 5:** Responsiveness from baseline to week 54

	***Group*: Patients with a > = 5 letter improvement on the ETDRS in their study eye between baseline and week 54**						
	**VFQ-25 Change Score**					**Guyatt’s Responsiveness**	
**VFQ-25 Domain**	**n**	**Mean**	***P *value from one-sample *t*-test**	Effect Size*	**SRM**^**†**^	**n**	**Statistic**^**‡**^
1. General Health	93	3.23	.14	0.15	0.15	30	0.12
2. General Vision	93	10.32	<.001	0.55	0.56	30	0.57
3. Ocular Pain	93	2.96	.16	0.13	0.15	30	0.14
4. Near Activities	93	5.69	.011	0.23	0.27	30	0.29
5. Distance Activities	93	3.67	.12	0.15	0.16	30	0.21
6. Social Functioning	93	0.54	.75	0.02	0.03	30	0.02
7. Mental Health	93	4.64	.024	0.17	0.24	30	0.21
8. Role Difficulties	93	11.29	<.001	0.41	0.38	30	0.49
9. Dependency	93	2.78	.25	0.09	0.12	30	0.10
10. Driving	56	5.73	.018	0.16	0.33	17	0.25
11. Colour Vision	91	1.92	.35	0.09	0.10	30	0.09
12. Peripheral Vision	93	4.30	.070	0.17	0.19	30	0.19
VFQ-25 Composite	93	4.90	<.001	0.26	0.43	30	0.39

* Effect size: the mean change score divided by the standard deviation of the baseline score.

† Standardized response mean: the mean change score divided by the standard deviation of the change score.

‡ Guyatt's responsiveness statistic: the mean change score divided by the standard deviation of change in subjects who remained stable. Stable groups are defined as an increase or a decrease of up to 1 letter.

There was also a degree of variation between the dimensions in terms of different statistical approaches. However, consistent with the mean change score, the same scales were shown to be responsive (except for Social Functioning) in terms of estimates of effect size, standardized response mean, and Guyatt's statistic at 54 weeks.

### MID

Table 6 provides estimates of MID using a distribution approach. MID ranged from 8.80 (General Vision) to 14.40 (Role Difficulties) with an overall 6.13 points for the VFQ-25 composite score using Â½ SD distribution based methods. SEM was available for eight out of 11 dimensions, due to the requirement of needing Cronbach’s alpha in the calculation. SEM showed similar results ranging from 8.79 (Driving) to 14.04 (Role Difficulties), with a lower estimate for the VFQ-25 composite score (3.33).

**Table 6:** Minimal important difference from baseline to week 54

**VFQ-25 Domain**	**N**	SEM*	**1/2 SD**
1. General Health	197	NA	10.95
2. General Vision	197	NA	8.80
3. Ocular Pain	197	11.98	10.53
4. Near Activities	197	9.17	10.24
5. Distance Activities	197	10.19	11.07
6. Social Functioning	197	9.64	9.87
7. Mental Health	197	10.21	10.56
8. Role Difficulties	197	14.04	14.40
9. Dependency	197	10.21	12.49
10. Driving	118	8.79	9.44
11. Colour Vision	194	NA	10.22
12. Peripheral Vision	196	NA	12.12
VFQ-25 Composite	197	3.33	6.13

* Standard deviation * √ (1- Cronbach's Alpha).

## Discussion

The analyses of the domain structures of the VFQ-25 do not perfectly describe the proposed structure of the instrument. The analyses provided support for the conceptual model but there were also some differences of note. The ‘worry’ item (Q 3) did not load onto the mental health domain in the new model, but instead became a separate and distinct single item factor. The general driving question was associated with social functioning whereas the driving in difficult conditions or at night items both fell out as a separate domain. The social functioning domain seemed to disappear with both items being redistributed to other domains. The near vision item which refers to seeing things on a crowded shelf was aligned with the distance vision domain as opposed to the near vision domain of the original model. The results were similar when using baseline data alone and when pooling baseline and follow-up data at Week 54. However, variable clustering methodology showed that none of the eight established multi-item scales met the criterion for further splitting.

The analyses of the instrument’s reliability were generally supportive. However internal consistency of the measure is often very restricted because many domains only include a single item. This lack of sufficient numbers of items is an important limitation in the design of the VFQ-25. It is better practice to have multiple items per domain, for example, between four and ten items as this provides a much more thorough assessment of the dimension. The overall measurement properties of the VFQ-25 (i.e. not just reliability) would be improved if there were more items per domain, or perhaps fewer domains.

The evidence to support concurrent and construct (discriminant and convergent) validity was mixed. Significant correlations were found between VFQ-25 and EQ-5D VAS scores, but these were generally low. Regression analyses found that different domains of the VFQ-25 were significantly predicted by variables such as ECOG, HbA1c, and ETDRS but this pattern of findings was not consistent. Indeed it wasn’t clear to the authors what pattern of results would necessarily be predicted. It’s interesting to note that based upon very similar patterns of correlations between visual acuity and VFQ-25 in patients with diabetic retinopathy, Matza et al. [35] concluded that the instrument “demonstrated construct validity”. The interpretation of whether something is psychometrically valid is subjective and reflects different points of view.

The analysis of known groups validity did demonstrate that the measure could differentiate between patients based on ETDRS letter score. However, the exploration of the validity of the VFQ-25 was rather complicated because of the wide conceptual scope of the instrument. The VFQ-25 includes domains which reflect central visual function – such as near and distance activities, and driving- domains which should be related to visual acuity in DME patients. The instrument also includes more general domains such as role difficulties and social functioning which will be influenced by many factors other than central vision. Therefore there are *a priori* reasons as to why these domains would not be related to ETDRS score. In reality, however, the association between domain scores and visual acuity was no greater for vision-related domains than it was for the more general domains. This indicates that much of what the VFQ-25 is measuring is not related to visual functioning. This argument is supported when the correlations with EQ-5D VAS are considered.

Other authors have raised fundamental concerns regarding the performance of the VFQ-25. Marella et al. [36] report a study which employed Rasch analysis to explore the performance of the VFQ-25 in patients with low vision and concluded the 12 factor structure of the VFQ-12 has no psychometric validity. They proposed a simpler two factor solution reflecting visual and socio-emotional functioning. Some of the items do not fit this structure and the authors suggest that these be dropped (when the instrument is used in patients with low vision). In addition the Rasch analysis indicated that many of the items could effectively be reduced to a dichotomous outcome. This study, and others [37,38], have also highlighted limitations with the VFQ-25. On balance the VFQ-25 has design flaws and failings related to its psychometric performance; nonetheless, it was able to measure various aspects of HRQoL in patients with DME.

There are some important limitations with our study. The analyses were undertaken retrospectively on clinical trial data and, consequently, the data available for the analyses were limited. Quite detailed ophthalmology data were available but there were limited data from other measures of HRQoL as might be expected in a clinical trial, as trial participants may already be overburdened with clinical measurements required by the protocol. HRQoL was restricted to the EQ-5D, and for these analyses only the VAS was used. The VAS is an assessment of general health status and as such was probably influenced by many factors. It would be useful to gather more detailed information perhaps using a generic profile measure of HRQoL, and in addition an alternative measure of vision-specific HRQoL. In our analyses of construct validity we relied on the EQ-5D VAS to benchmark the VFQ-25 data against. We believe this is a limitation of our study because the EQ-5D VAS is a single item scale which assesses self-rated health. Therefore, while the EQ-5D VAS may be able to provide an overview of health status, it is not specific to the domains that the VFQ-25 is measuring. This makes it difficult to interpret the importance of correlations. It would have been preferable to benchmark against another profile measure. In addition, we used the EQ-5D VAS to benchmark MID of vision specific functioning and HRQoL. While estimates of MID on these measures have been previously reported, it should be remembered that the EQ-5D VAS assesses overall health status. Therefore this is not the most appropriate anchor to use for assessing the MID of vision-specific functioning and HRQL as measured by the VFQ-25. It would have been preferable to use anchors that are much more specific to the domain being assessed, but such data were not captured in the trial.

It should also be noted that because of the nature of the condition, the data were collected using a validated telephone interview script rather than through self-report or face-to-face interviews, apart from in India where telephone interviews were not possible. This administration method was not specifically examined as a predictor variable in the psychometric analyses.

The present study suggests that the VFQ-25 has some validity as a measure of HRQoL in patients with DME. It performs as well in DME as in assessment of diabetic retinopathy [10], and age related macular degeneration [39]. However, consistent with other studies, the VFQ-25 has some important limitations. To improve measurement of vision-related quality of life in DME patients, a new or modified instrument should be developed.

## Competing interests

A.J. Lloyd is an employee of Oxford Outcomes, an ICON company, who was a paid consultant to Pfizer in connection with the development of this manuscript. M. Turner and G. Lai are employees of ICON and were paid consultants to Pfizer in connection with the development of this manuscript. J. Loftus and A. Pleil are employees of Pfizer, the sponsor of the study.

## Authors’ contributions

All authors, AJL, JL, MT, GL, and AP, participated in developing the concept, obtaining and analysing the data, interpreting the data, drafting and revising the manuscript, and approving the final version being submitted for review and publication. All authors read and approved the final manuscript.
